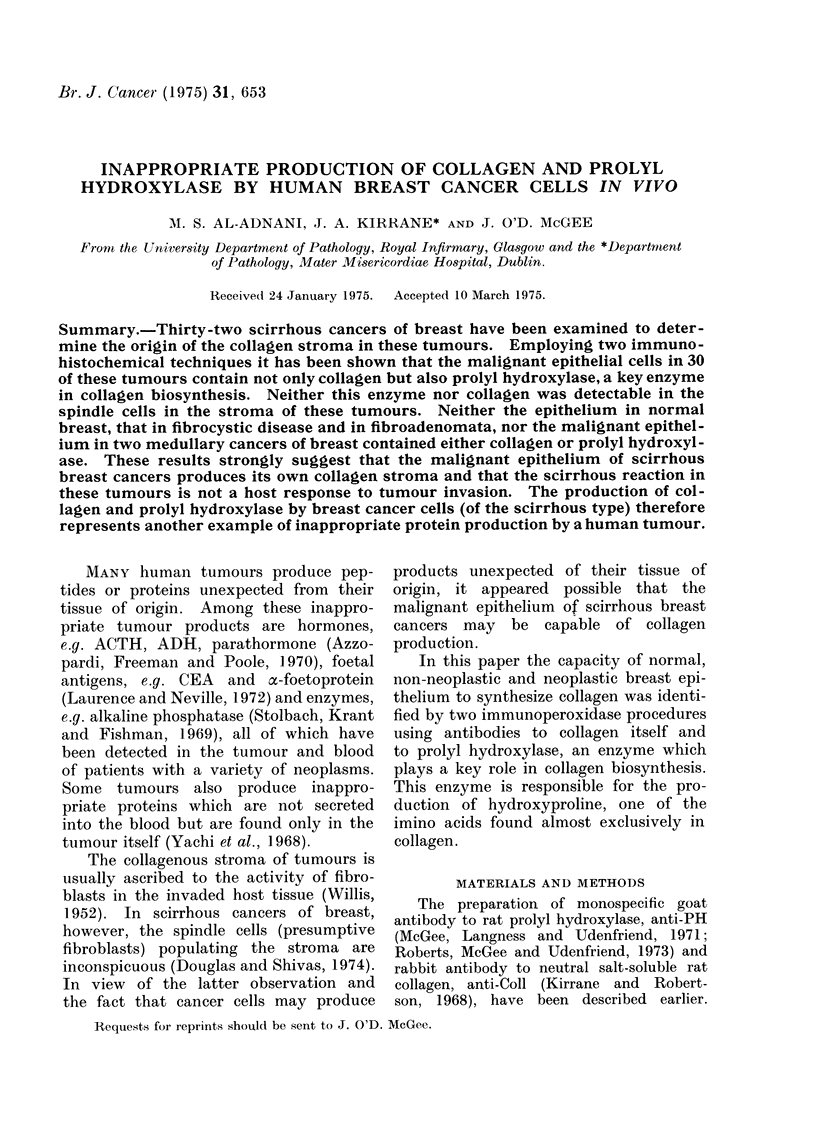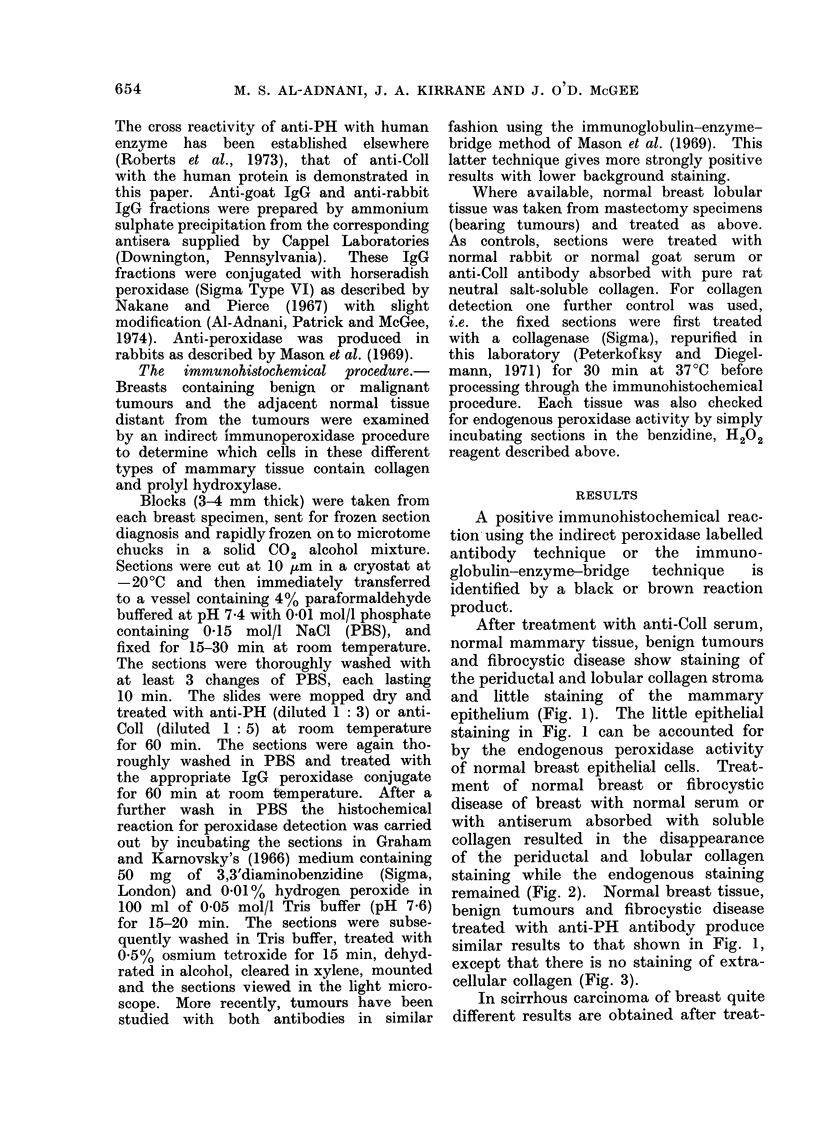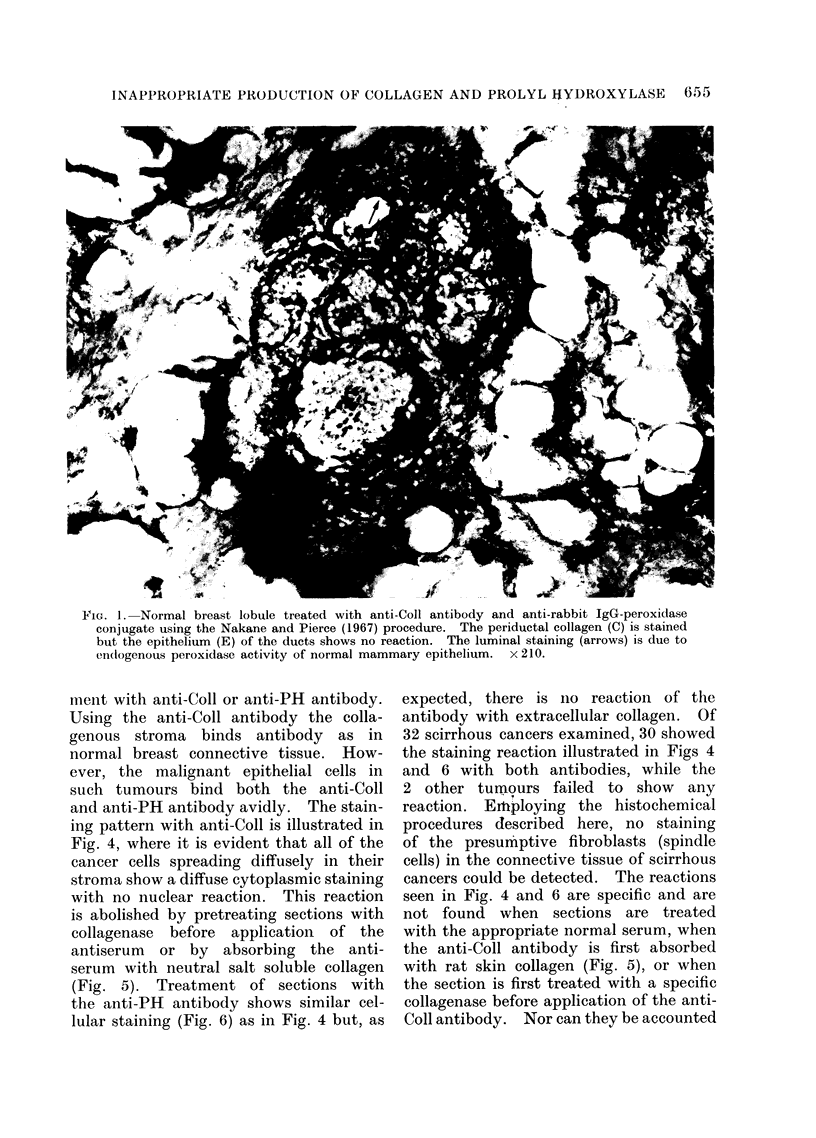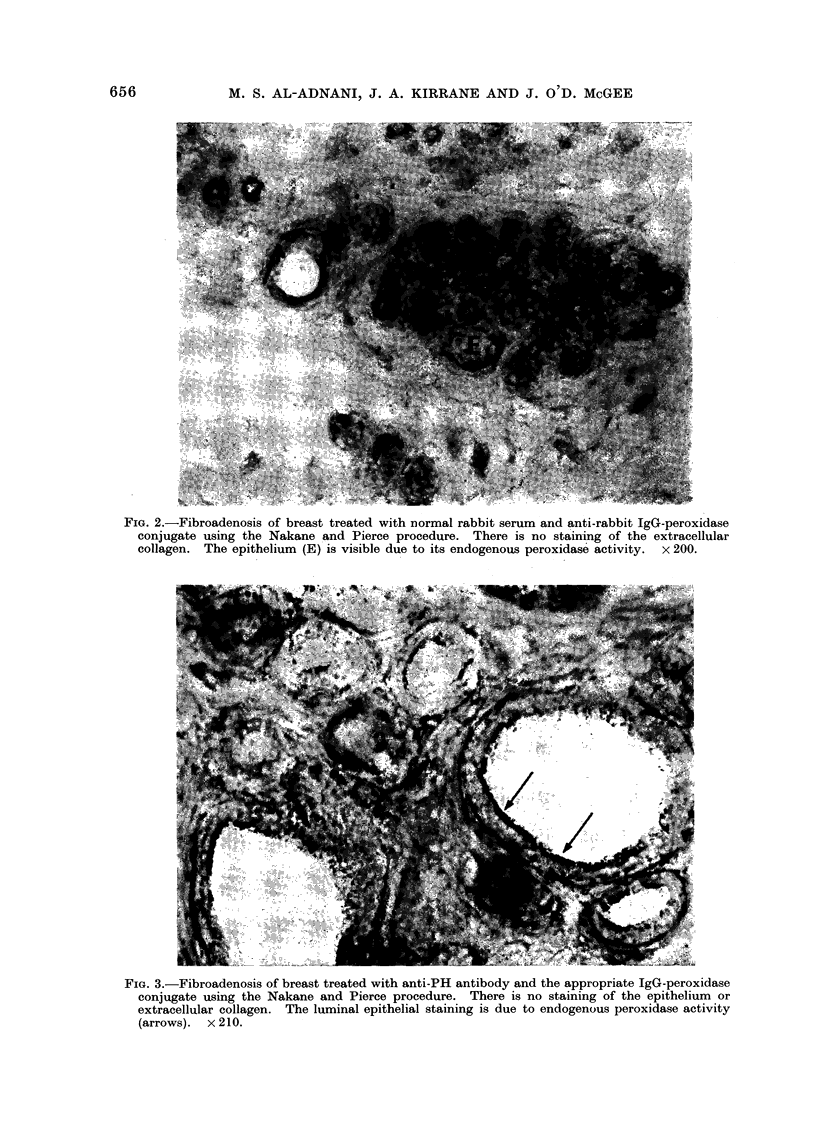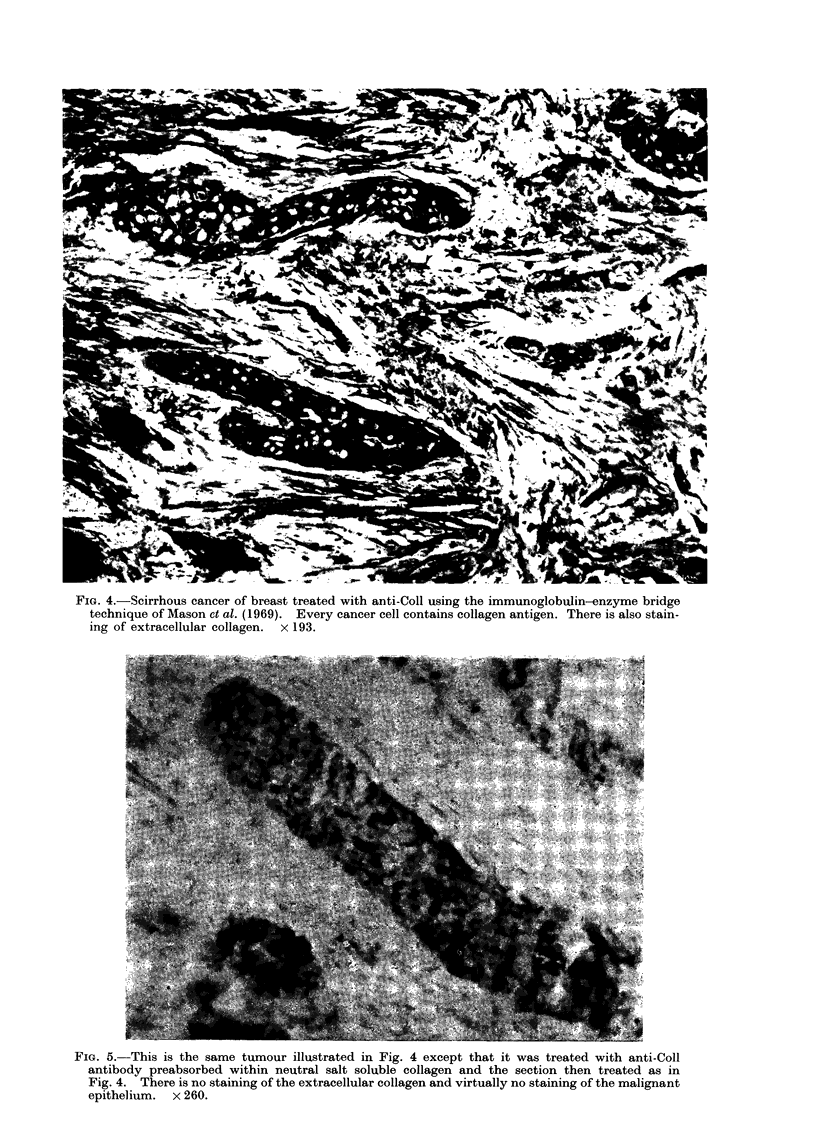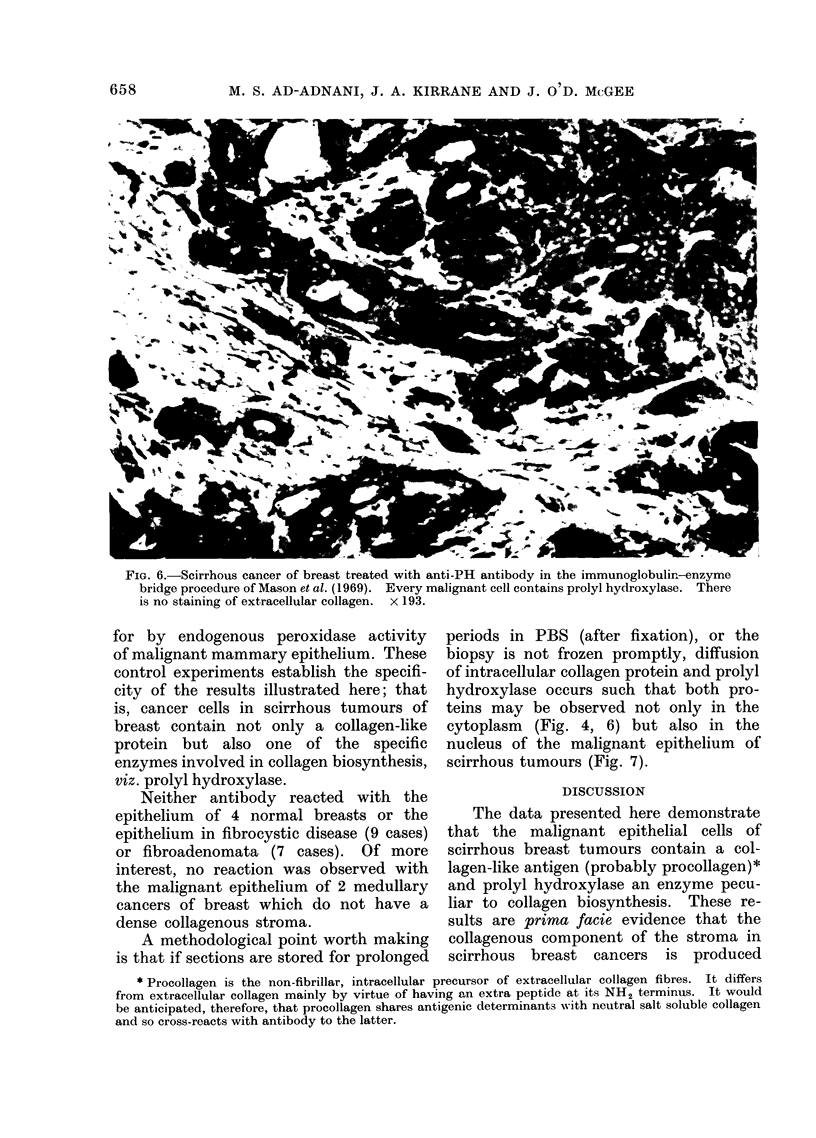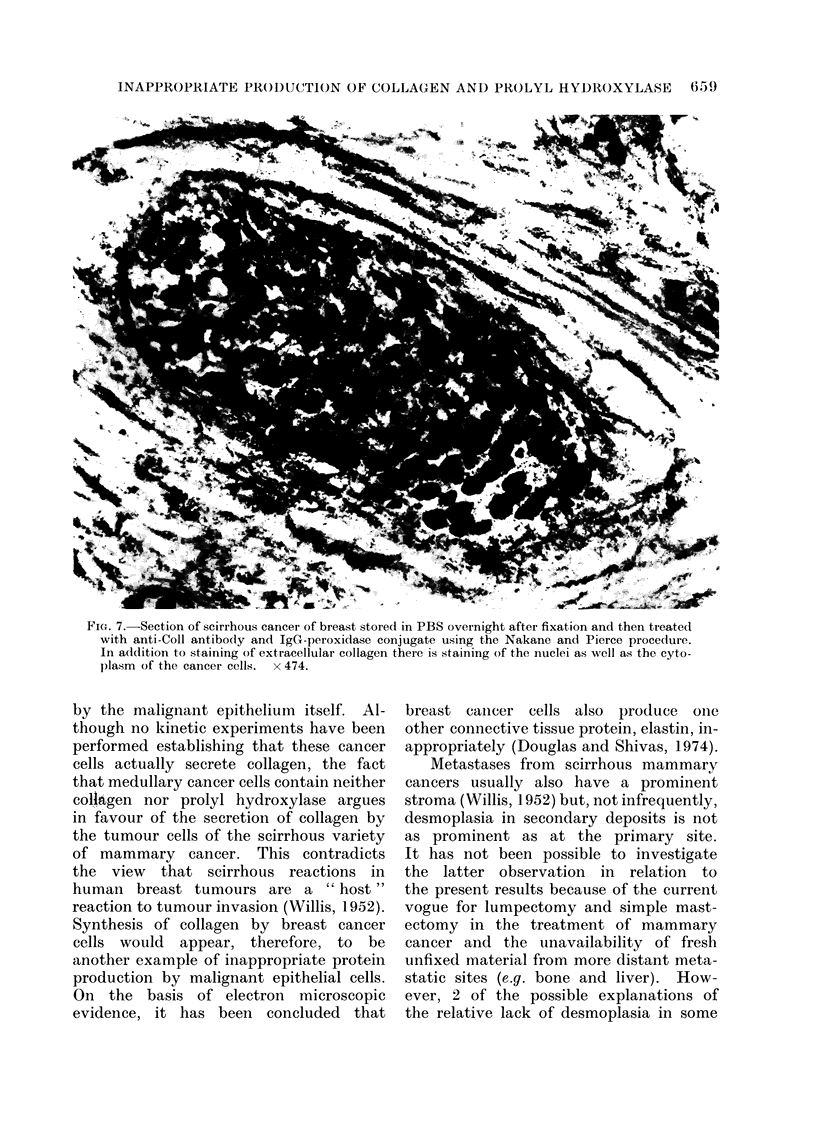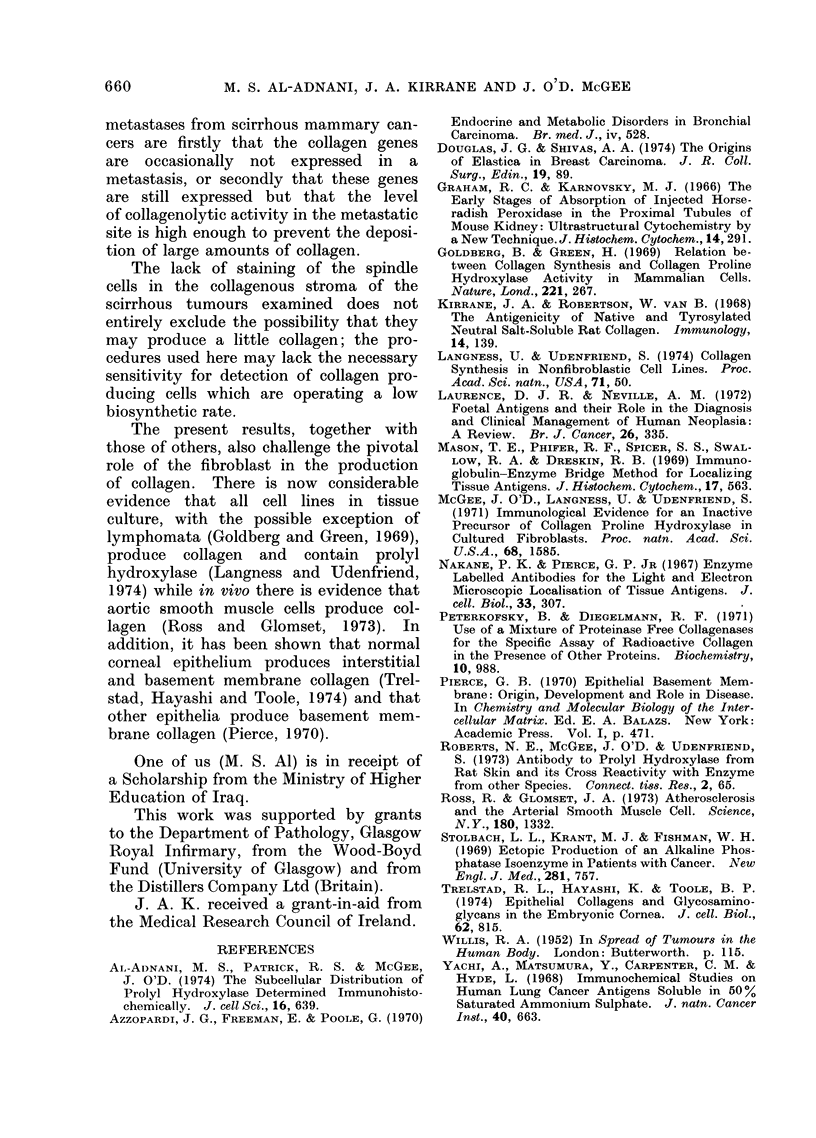# Inappropriate production of collagen and prolyl hydroxylase by human breast cancer cells in vivo.

**DOI:** 10.1038/bjc.1975.112

**Published:** 1975-06

**Authors:** M. S. Al-Adnani, J. A. Kirrane, J. O. McGee

## Abstract

**Images:**


*Br. J. Cancer* (1975) **31**, 653

### INAPPROPRIATE PRODUCTION OF COLLAGEN AND PROLYL HYDROXYLASE BY HUMAN BREAST CANCER CELLS *IN VIVO*

M. S. AL-ADNANI, J. A. KIRRANE* AND J. O'D. McGEE

*From the University Department of Pathology, Royal Infirmary, Glasgow and the *Department of Pathology, Mater Misericordiae Hospital, Dublin.*

Received 24 January 1975. Accepted 10 March 1975.

**Summary.—Thirty-two scirrhous cancers of breast have been examined to determine the origin of the collagen stroma in these tumours. Employing two immunohistochemical techniques it has been shown that the malignant epithelial cells in 30 of these tumours contain not only collagen but also prolyl hydroxylase, a key enzyme in collagen biosynthesis. Neither this enzyme nor collagen was detectable in the spindle cells in the stroma of these tumours. Neither the epithelium in normal breast, that in fibrocystic disease and in fibroadenomata, nor the malignant epithelium in two medullary cancers of breast contained either collagen or prolyl hydroxylase. These results strongly suggest that the malignant epithelium of scirrhous breast cancers produces its own collagen stroma and that the scirrhous reaction in these tumours is not a host response to tumour invasion. The production of collagen and prolyl hydroxylase by breast cancer cells (of the scirrhous type) therefore represents another example of inappropriate protein production by a human tumour.**

MANY human tumours produce peptides or proteins unexpected from their tissue of origin. Among these inappropriate tumour products are hormones, *e.g.* ACTH, ADH, parathormone (Azzopardi, Freeman and Poole, 1970), foetal antigens, *e.g.* CEA and α-foetoprotein (Laurence and Neville, 1972) and enzymes, *e.g.* alkaline phosphatase (Stolbach, Krant and Fishman, 1969), all of which have been detected in the tumour and blood of patients with a variety of neoplasms. Some tumours also produce inappropriate proteins which are not secreted into the blood but are found only in the tumour itself (Yachi *et al.*, 1968).

The collagenous stroma of tumours is usually ascribed to the activity of fibroblasts in the invaded host tissue (Willis, 1952). In scirrhous cancers of breast, however, the spindle cells (presumptive fibroblasts) populating the stroma are inconspicuous (Douglas and Shivas, 1974). In view of the latter observation and the fact that cancer cells may produce products unexpected of their tissue of origin, it appeared possible that the malignant epithelium of scirrhous breast cancers may be capable of collagen production.

In this paper the capacity of normal, non-neoplastic and neoplastic breast epithelium to synthesize collagen was identified by two immunoperoxidase procedures using antibodies to collagen itself and to prolyl hydroxylase, an enzyme which plays a key role in collagen biosynthesis. This enzyme is responsible for the production of hydroxyproline, one of the imino acids found almost exclusively in collagen.

### MATERIALS AND METHODS

The preparation of monospecific goat antibody to rat prolyl hydroxylase, anti-PH (McGee, Langness and Udenfriend, 1971; Roberts, McGee and Udenfriend, 1973) and rabbit antibody to neutral salt-soluble rat collagen, anti-Coll (Kirrane and Robertson, 1968), have been described earlier.

Requests for reprints should be sent to J. O'D. McGee.

The cross reactivity of anti-PH with human enzyme has been established elsewhere (Roberts *et al.*, 1973), that of anti-Coll with the human protein is demonstrated in this paper. Anti-goat IgG and anti-rabbit IgG fractions were prepared by ammonium sulphate precipitation from the corresponding antisera supplied by Cappel Laboratories (Downington, Pennsylvania). These IgG fractions were conjugated with horseradish peroxidase (Sigma Type VI) as described by Nakane and Pierce (1967) with slight modification (Al-Adnani, Patrick and McGee, 1974). Anti-peroxidase was produced in rabbits as described by Mason *et al.* (1969).

*The immunohistochemical procedure.*—Breasts containing benign or malignant tumours and the adjacent normal tissue distant from the tumours were examined by an indirect immunoperoxidase procedure to determine which cells in these different types of mammary tissue contain collagen and prolyl hydroxylase.

Blocks (3–4 mm thick) were taken from each breast specimen, sent for frozen section diagnosis and rapidly frozen on to microtome chucks in a solid $CO_2$ alcohol mixture. Sections were cut at 10 $\mu$m in a cryostat at $-20°C$ and then immediately transferred to a vessel containing 4% paraformaldehyde buffered at pH 7·4 with 0·01 mol/l phosphate containing 0·15 mol/l NaCl (PBS), and fixed for 15–30 min at room temperature. The sections were thoroughly washed with at least 3 changes of PBS, each lasting 10 min. The slides were mopped dry and treated with anti-PH (diluted 1 : 3) or anti-Coll (diluted 1 : 5) at room temperature for 60 min. The sections were again thoroughly washed in PBS and treated with the appropriate IgG peroxidase conjugate for 60 min at room temperature. After a further wash in PBS the histochemical reaction for peroxidase detection was carried out by incubating the sections in Graham and Karnovsky's (1966) medium containing 50 mg of 3,3'diaminobenzidine (Sigma, London) and 0·01% hydrogen peroxide in 100 ml of 0·05 mol/l Tris buffer (pH 7·6) for 15–20 min. The sections were subsequently washed in Tris buffer, treated with 0·5% osmium tetroxide for 15 min, dehydrated in alcohol, cleared in xylene, mounted and the sections viewed in the light microscope. More recently, tumours have been studied with both antibodies in similar fashion using the immunoglobulin–enzyme–bridge method of Mason *et al.* (1969). This latter technique gives more strongly positive results with lower background staining.

Where available, normal breast lobular tissue was taken from mastectomy specimens (bearing tumours) and treated as above. As controls, sections were treated with normal rabbit or normal goat serum or anti-Coll antibody absorbed with pure rat neutral salt-soluble collagen. For collagen detection one further control was used, *i.e.* the fixed sections were first treated with a collagenase (Sigma), repurified in this laboratory (Peterkofksy and Diegelmann, 1971) for 30 min at 37°C before processing through the immunohistochemical procedure. Each tissue was also checked for endogenous peroxidase activity by simply incubating sections in the benzidine, $H_2O_2$ reagent described above.

### RESULTS

A positive immunohistochemical reaction using the indirect peroxidase labelled antibody technique or the immunoglobulin–enzyme–bridge technique is identified by a black or brown reaction product.

After treatment with anti-Coll serum, normal mammary tissue, benign tumours and fibrocystic disease show staining of the periductal and lobular collagen stroma and little staining of the mammary epithelium (Fig. 1). The little epithelial staining in Fig. 1 can be accounted for by the endogenous peroxidase activity of normal breast epithelial cells. Treatment of normal breast or fibrocystic disease of breast with normal serum or with antiserum absorbed with soluble collagen resulted in the disappearance of the periductal and lobular collagen staining while the endogenous staining remained (Fig. 2). Normal breast tissue, benign tumours and fibrocystic disease treated with anti-PH antibody produce similar results to that shown in Fig. 1, except that there is no staining of extracellular collagen (Fig. 3).

In scirrhous carcinoma of breast quite different results are obtained after treat-

FIG. 1.—Normal breast lobule treated with anti-Coll antibody and anti-rabbit IgG-peroxidase conjugate using the Nakane and Pierce (1967) procedure. The periductal collagen (C) is stained but the epithelium (E) of the ducts shows no reaction. The luminal staining (arrows) is due to endogenous peroxidase activity of normal mammary epithelium. ×210.

ment with anti-Coll or anti-PH antibody. Using the anti-Coll antibody the collagenous stroma binds antibody as in normal breast connective tissue. However, the malignant epithelial cells in such tumours bind both the anti-Coll and anti-PH antibody avidly. The staining pattern with anti-Coll is illustrated in Fig. 4, where it is evident that all of the cancer cells spreading diffusely in their stroma show a diffuse cytoplasmic staining with no nuclear reaction. This reaction is abolished by pretreating sections with collagenase before application of the antiserum or by absorbing the antiserum with neutral salt soluble collagen (Fig. 5). Treatment of sections with the anti-PH antibody shows similar cellular staining (Fig. 6) as in Fig. 4 but, as expected, there is no reaction of the antibody with extracellular collagen. Of 32 scirrhous cancers examined, 30 showed the staining reaction illustrated in Figs 4 and 6 with both antibodies, while the 2 other tumours failed to show any reaction. Employing the histochemical procedures described here, no staining of the presumptive fibroblasts (spindle cells) in the connective tissue of scirrhous cancers could be detected. The reactions seen in Fig. 4 and 6 are specific and are not found when sections are treated with the appropriate normal serum, when the anti-Coll antibody is first absorbed with rat skin collagen (Fig. 5), or when the section is first treated with a specific collagenase before application of the anti-Coll antibody. Nor can they be accounted

FIG. 2.—Fibroadenosis of breast treated with normal rabbit serum and anti-rabbit IgG-peroxidase conjugate using the Nakane and Pierce procedure. There is no staining of the extracellular collagen. The epithelium (E) is visible due to its endogenous peroxidase activity. ×200.

FIG. 3.—Fibroadenosis of breast treated with anti-PH antibody and the appropriate IgG-peroxidase conjugate using the Nakane and Pierce procedure. There is no staining of the epithelium or extracellular collagen. The luminal epithelial staining is due to endogenous peroxidase activity (arrows). ×210.

FIG. 4.—Scirrhous cancer of breast treated with anti-Coll using the immunoglobulin–enzyme bridge technique of Mason *et al.* (1969). Every cancer cell contains collagen antigen. There is also staining of extracellular collagen. ×193.

FIG. 5.—This is the same tumour illustrated in Fig. 4 except that it was treated with anti-Coll antibody preabsorbed within neutral salt soluble collagen and the section then treated as in Fig. 4. There is no staining of the extracellular collagen and virtually no staining of the malignant epithelium. ×260.

FIG. 6.—Scirrhous cancer of breast treated with anti-PH antibody in the immunoglobulin–enzyme bridge procedure of Mason *et al.* (1969). Every malignant cell contains prolyl hydroxylase. There is no staining of extracellular collagen. ×193.

for by endogenous peroxidase activity of malignant mammary epithelium. These control experiments establish the specificity of the results illustrated here; that is, cancer cells in scirrhous tumours of breast contain not only a collagen-like protein but also one of the specific enzymes involved in collagen biosynthesis, *viz.* prolyl hydroxylase.

Neither antibody reacted with the epithelium of 4 normal breasts or the epithelium in fibrocystic disease (9 cases) or fibroadenomata (7 cases). Of more interest, no reaction was observed with the malignant epithelium of 2 medullary cancers of breast which do not have a dense collagenous stroma.

A methodological point worth making is that if sections are stored for prolonged periods in PBS (after fixation), or the biopsy is not frozen promptly, diffusion of intracellular collagen protein and prolyl hydroxylase occurs such that both proteins may be observed not only in the cytoplasm (Fig. 4, 6) but also in the nucleus of the malignant epithelium of scirrhous tumours (Fig. 7).

### DISCUSSION

The data presented here demonstrate that the malignant epithelial cells of scirrhous breast tumours contain a collagen-like antigen (probably procollagen)* and prolyl hydroxylase an enzyme peculiar to collagen biosynthesis. These results are *prima facie* evidence that the collagenous component of the stroma in scirrhous breast cancers is produced

* Procollagen is the non-fibrillar, intracellular precursor of extracellular collagen fibres. It differs from extracellular collagen mainly by virtue of having an extra peptide at its $NH_2$ terminus. It would be anticipated, therefore, that procollagen shares antigenic determinants with neutral salt soluble collagen and so cross-reacts with antibody to the latter.

FIG. 7.—Section of scirrhous cancer of breast stored in PBS overnight after fixation and then treated with anti-Coll antibody and IgG-peroxidase conjugate using the Nakane and Pierce procedure. In addition to staining of extracellular collagen there is staining of the nuclei as well as the cytoplasm of the cancer cells. ×474.

by the malignant epithelium itself. Although no kinetic experiments have been performed establishing that these cancer cells actually secrete collagen, the fact that medullary cancer cells contain neither collagen nor prolyl hydroxylase argues in favour of the secretion of collagen by the tumour cells of the scirrhous variety of mammary cancer. This contradicts the view that scirrhous reactions in human breast tumours are a "host" reaction to tumour invasion (Willis, 1952). Synthesis of collagen by breast cancer cells would appear, therefore, to be another example of inappropriate protein production by malignant epithelial cells. On the basis of electron microscopic evidence, it has been concluded that breast cancer cells also produce one other connective tissue protein, elastin, inappropriately (Douglas and Shivas, 1974).

Metastases from scirrhous mammary cancers usually also have a prominent stroma (Willis, 1952) but, not infrequently, desmoplasia in secondary deposits is not as prominent as at the primary site. It has not been possible to investigate the latter observation in relation to the present results because of the current vogue for lumpectomy and simple mastectomy in the treatment of mammary cancer and the unavailability of fresh unfixed material from more distant metastatic sites (*e.g.* bone and liver). However, 2 of the possible explanations of the relative lack of desmoplasia in some metastases from scirrhous mammary cancers are firstly that the collagen genes are occasionally not expressed in a metastasis, or secondly that these genes are still expressed but that the level of collagenolytic activity in the metastatic site is high enough to prevent the deposition of large amounts of collagen.

The lack of staining of the spindle cells in the collagenous stroma of the scirrhous tumours examined does not entirely exclude the possibility that they may produce a little collagen; the procedures used here may lack the necessary sensitivity for detection of collagen producing cells which are operating a low biosynthetic rate.

The present results, together with those of others, also challenge the pivotal role of the fibroblast in the production of collagen. There is now considerable evidence that all cell lines in tissue culture, with the possible exception of lymphomata (Goldberg and Green, 1969), produce collagen and contain prolyl hydroxylase (Langness and Udenfriend, 1974) while *in vivo* there is evidence that aortic smooth muscle cells produce collagen (Ross and Glomset, 1973). In addition, it has been shown that normal corneal epithelium produces interstitial and basement membrane collagen (Trelstad, Hayashi and Toole, 1974) and that other epithelia produce basement membrane collagen (Pierce, 1970).

One of us (M. S. Al) is in receipt of a Scholarship from the Ministry of Higher Education of Iraq.

This work was supported by grants to the Department of Pathology, Glasgow Royal Infirmary, from the Wood-Boyd Fund (University of Glasgow) and from the Distillers Company Ltd (Britain).

J. A. K. received a grant-in-aid from the Medical Research Council of Ireland.

### REFERENCES

Al-Adnani, M. S., Patrick, R. S. & McGee, J. O'D. (1974) The Subcellular Distribution of Prolyl Hydroxylase Determined Immunohistochemically. *J. cell Sci.*, **16**, 639.

Azzopardi, J. G., Freeman, E. & Poole, G. (1970) Endocrine and Metabolic Disorders in Bronchial Carcinoma. *Br. med. J.*, iv, 528.

Douglas, J. G. & Shivas, A. A. (1974) The Origins of Elastica in Breast Carcinoma. *J. R. Coll. Surg., Edin.*, **19**, 89.

Graham, R. C. & Karnovsky, M. J. (1966) The Early Stages of Absorption of Injected Horseradish Peroxidase in the Proximal Tubules of Mouse Kidney: Ultrastructural Cytochemistry by a New Technique. *J. Histochem. Cytochem.*, **14**, 291.

Goldberg, B. & Green, H. (1969) Relation between Collagen Synthesis and Collagen Proline Hydroxylase Activity in Mammalian Cells. *Nature, Lond.*, **221**, 267.

Kirrane, J. A. & Robertson, W. van B. (1968) The Antigenicity of Native and Tyrosylated Neutral Salt-Soluble Rat Collagen. *Immunology*, **14**, 139.

Langness, U. & Udenfriend, S. (1974) Collagen Synthesis in Nonfibroblastic Cell Lines. *Proc. Acad. Sci. natn., USA*, **71**, 50.

Laurence, D. J. R. & Neville, A. M. (1972) Foetal Antigens and their Role in the Diagnosis and Clinical Management of Human Neoplasia: A Review. *Br. J. Cancer*, **26**, 335.

Mason, T. E., Phifer, R. F., Spicer, S. S., Swallow, R. A. & Dreskin, R. B. (1969) Immunoglobulin–Enzyme Bridge Method for Localizing Tissue Antigens. *J. Histochem. Cytochem.*, **17**, 563.

McGee, J. O'D., Langness, U. & Udenfriend, S. (1971) Immunological Evidence for an Inactive Precursor of Collagen Proline Hydroxylase in Cultured Fibroblasts. *Proc. natn. Acad. Sci. U.S.A.*, **68**, 1585.

Nakane, P. K. & Pierce, G. P. Jr (1967) Enzyme Labelled Antibodies for the Light and Electron Microscopic Localisation of Tissue Antigens. *J. cell. Biol.*, **33**, 307.

Peterkofsky, B. & Diegelmann, R. F. (1971) Use of a Mixture of Proteinase Free Collagenases for the Specific Assay of Radioactive Collagen in the Presence of Other Proteins. *Biochemistry*, **10**, 988.

Pierce, G. B. (1970) Epithelial Basement Membrane: Origin, Development and Role in Disease. In *Chemistry and Molecular Biology of the Intercellular Matrix*. Ed. E. A. Balazs. New York: Academic Press. Vol. I, p. 471.

Roberts, N. E., McGee, J. O'D. & Udenfriend, S. (1973) Antibody to Prolyl Hydroxylase from Rat Skin and its Cross Reactivity with Enzyme from other Species. *Connect. tiss. Res.*, **2**, 65.

Ross, R. & Glomset, J. A. (1973) Atherosclerosis and the Arterial Smooth Muscle Cell. *Science, N.Y.*, **180**, 1332.

Stolbach, L. L., Krant, M. J. & Fishman, W. H. (1969) Ectopic Production of an Alkaline Phosphatase Isoenzyme in Patients with Cancer. *New Engl. J. Med.*, **281**, 757.

Trelstad, R. L., Hayashi, K. & Toole, B. P. (1974) Epithelial Collagens and Glycosaminoglycans in the Embryonic Cornea. *J. cell. Biol.*, **62**, 815.

Willis, R. A. (1952) In *Spread of Tumours in the Human Body*. London: Butterworth. p. 115.

Yachi, A., Matsumura, Y., Carpenter, C. M. & Hyde, L. (1968) Immunochemical Studies on Human Lung Cancer Antigens Soluble in 50% Saturated Ammonium Sulphate. *J. natn. Cancer Inst.*, **40**, 663.